# The genome sequence of the White-point, *Mythimna albipuncta *(Denis & Schiffermüller, 1775)

**DOI:** 10.12688/wellcomeopenres.20682.1

**Published:** 2024-02-19

**Authors:** Douglas Boyes, Peter W. H. Holland, University of Oxford and Wytham Woods Genome Acquisition Lab, Darwin Tree of Life Barcoding collective, Wellcome Sanger Institute Tree of Life Management, Samples and Laboratory team, Wellcome Sanger Institute Scientific Operations: Sequencing Operations, Wellcome Sanger Institute Tree of Life Core Informatics team, Tree of Life Core Informatics collective, Darwin Tree of Life Consortium

**Affiliations:** 1 UK Centre for Ecology & Hydrology, Wallingford, England, UK; 2 Department of Biology, University of Oxford, Oxford, England, UK

**Keywords:** Mythimna albipuncta, white-point, genome sequence, chromosomal, Lepidoptera

## Abstract

We present a genome assembly from an individual male *Mythimna albipuncta* (the White-point; Arthropoda; Insecta; Lepidoptera; Noctuidae). The genome sequence is 698.6 megabases in span. Most of the assembly is scaffolded into 31 chromosomal pseudomolecules, including the Z sex chromosome. The mitochondrial genome has also been assembled and is 15.38 kilobases in length. Gene annotation of this assembly on Ensembl identified 13,679 protein coding genes.

## Species taxonomy

Eukaryota; Metazoa; Eumetazoa; Bilateria; Protostomia; Ecdysozoa; Panarthropoda; Arthropoda; Mandibulata; Pancrustacea; Hexapoda; Insecta; Dicondylia; Pterygota; Neoptera; Endopterygota; Amphiesmenoptera; Lepidoptera; Glossata; Neolepidoptera; Heteroneura; Ditrysia; Obtectomera; Noctuoidea; Noctuidae; Hadeninae; *Mythimna*; *Mythimna albipuncta* (Denis & Schiffermüller, 1775) (NCBI:txid987983).

## Background

*Mythimna albipuncta*, the White-point, is a moth in the family Noctuidae found across much of central and northern Europe, with scattered records from Ukraine, Estonia, Russia, Tunisia and Morocco ( GBIF Secretariat, 2023). The adult moth has ochreous-brown forewings with indistinct markings apart from a conspicuous white spot in the position of the reniform stigma. It can be distinguished from the similar Clay moth, *Mythimna farrago* ( Boyes *et al.*, 2022), by a less elongated forewing shape and by the white spot being either diamond-shaped or rounded.

Through most of the twentieth century, the species was not a resident breeding species in Britain, but was recorded as an infrequent immigrant species along the south and east coasts. There was evidence of a second brood produced by early summer migrant moths in southern counties such as Dorset ( Davey, 2009). In the past twenty years numbers of records have increased dramatically; for example, in Norfolk there were just 4 records in 2000, but this number increased to almost 4000 records in 2021 ( NorfolkMoths, 2023). The increase in recording frequency, seen in all southern counties of Britain, is attributed to widespread establishment as a resident breeding species, supplemented by ongoing influx of migrant individuals from France and Spain ( Davey, 2009).

The larvae of *M. albipuncta* feed on various species of grass including cock’s-foot ( *Dactylus glomerata*), overwintering at the larval stage. Hibernation is clearly not obligatory, as a second brood can occur in summer in Britain ( Davey, 2009) and in captivity adults can be reared from the egg in 2 to 3 months ( Heath & Emmet, 1983).

The genome sequence of *Mythimna albipuncta* was determined and assembled as part of the Darwin Tree of Life project. The complete genome sequence will contribute to the growing set of resources for studying molecular evolution in the Lepidoptera.

## Genome sequence report

**Figure 1.**
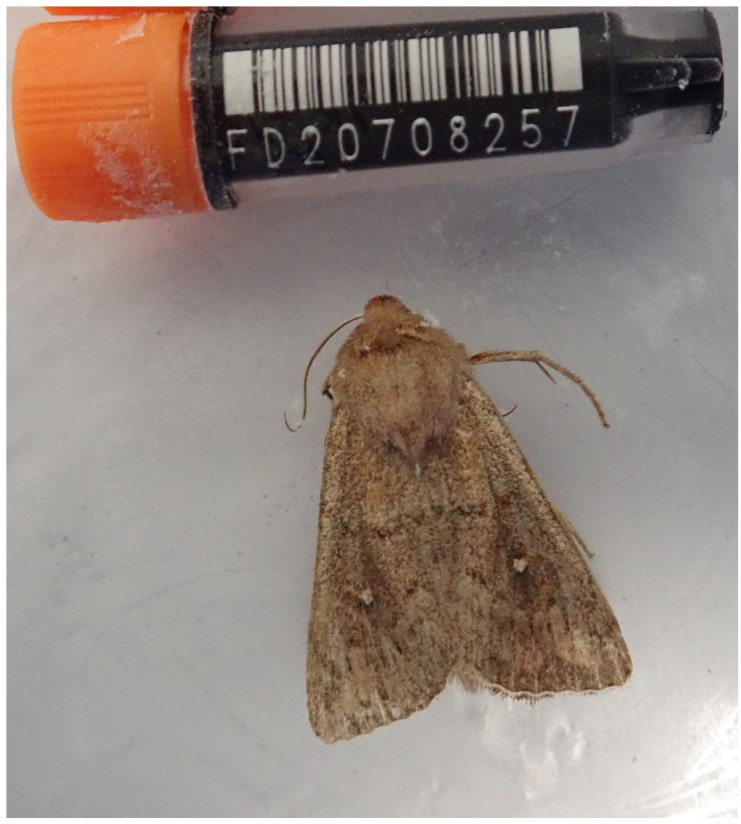
Photograph of the *Mythimna albipuncta* (ilMytAlbi1) specimen used for genome sequencing.

The genome was sequenced from one male *Mythimna albipuncta* ( Figure 1) collected from Wytham Woods, Oxfordshire, UK (51.77, –1.34). A total of 37-fold coverage in Pacific Biosciences single-molecule HiFi long reads and 57-fold coverage in 10X Genomics read clouds was generated. Primary assembly contigs were scaffolded with chromosome conformation Hi-C data. Manual assembly curation corrected 7 missing joins or mis-joins and removed one haplotypic duplication, reducing the scaffold number by 5.71%, and increasing the scaffold N50 by 0.44%.

**Figure 2.**
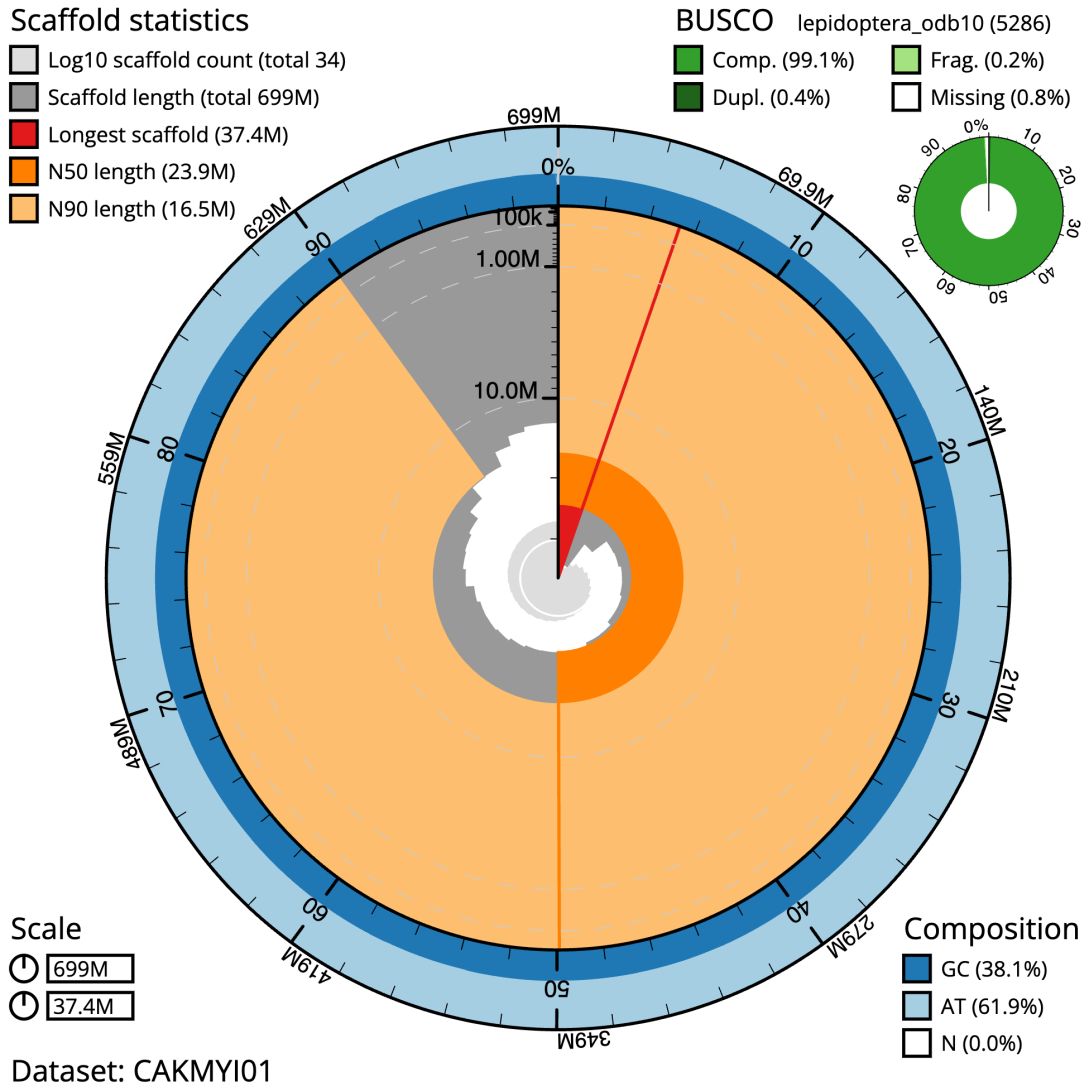
Genome assembly of *Mythimna albipuncta*, ilMytAlbi1.1: metrics. The BlobToolKit Snailplot shows N50 metrics and BUSCO gene completeness. The main plot is divided into 1,000 size-ordered bins around the circumference with each bin representing 0.1% of the 698,566,279 bp assembly. The distribution of scaffold lengths is shown in dark grey with the plot radius scaled to the longest scaffold present in the assembly (37,427,200 bp, shown in red). Orange and pale-orange arcs show the N50 and N90 scaffold lengths (23,908,972 and 16,462,175 bp), respectively. The pale grey spiral shows the cumulative scaffold count on a log scale with white scale lines showing successive orders of magnitude. The blue and pale-blue area around the outside of the plot shows the distribution of GC, AT and N percentages in the same bins as the inner plot. A summary of complete, fragmented, duplicated and missing BUSCO genes in the lepidoptera_odb10 set is shown in the top right. An interactive version of this figure is available at https://blobtoolkit.genomehubs.org/view/CAKMYI01/dataset/CAKMYI01/snail.

**Figure 3.**
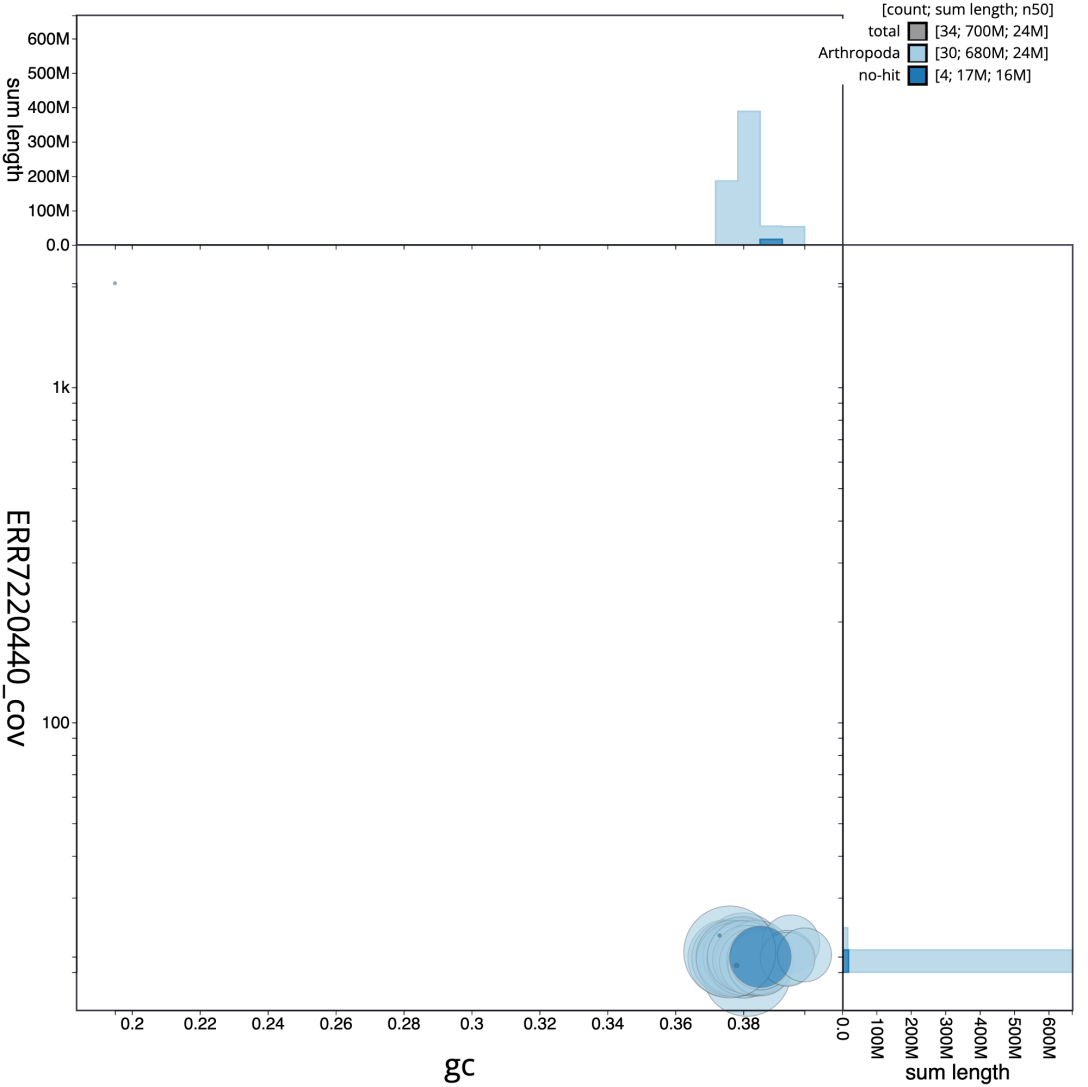
Genome assembly of *Mythimna albipuncta*, ilMytAlbi1.1: BlobToolKit GC-coverage plot. Scaffolds are coloured by phylum. Circles are sized in proportion to scaffold length. Histograms show the distribution of scaffold length sum along each axis. An interactive version of this figure is available at https://blobtoolkit.genomehubs.org/view/CAKMYI01/dataset/CAKMYI01/blob.

**Figure 4.**
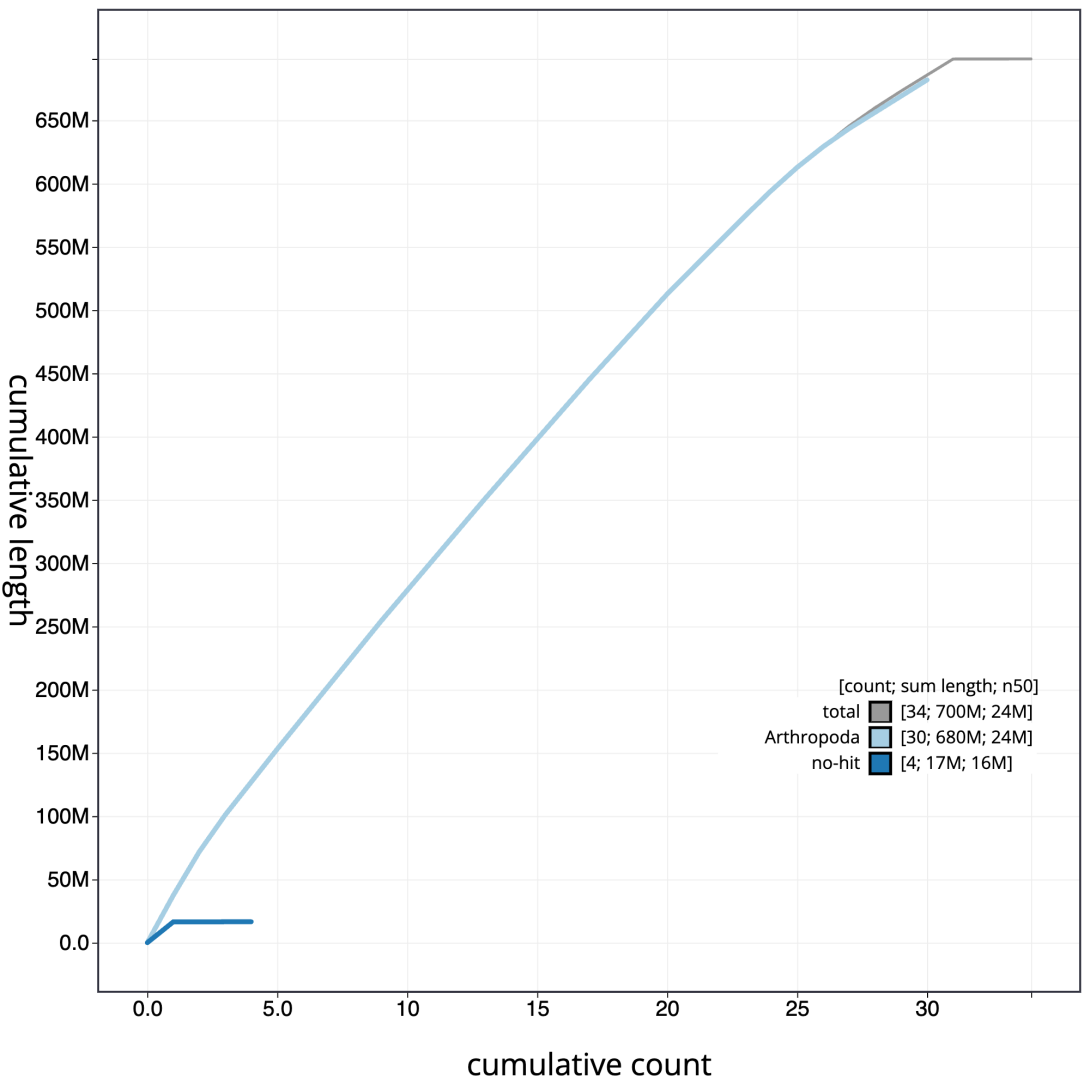
Genome assembly of *Mythimna albipuncta*, ilMytAlbi1.1: BlobToolKit cumulative sequence plot. The grey line shows cumulative length for all scaffolds. Coloured lines show cumulative lengths of scaffolds assigned to each phylum using the buscogenes taxrule. An interactive version of this figure is available at https://blobtoolkit.genomehubs.org/view/CAKMYI01/dataset/CAKMYI01/cumulative.

**Figure 5.**
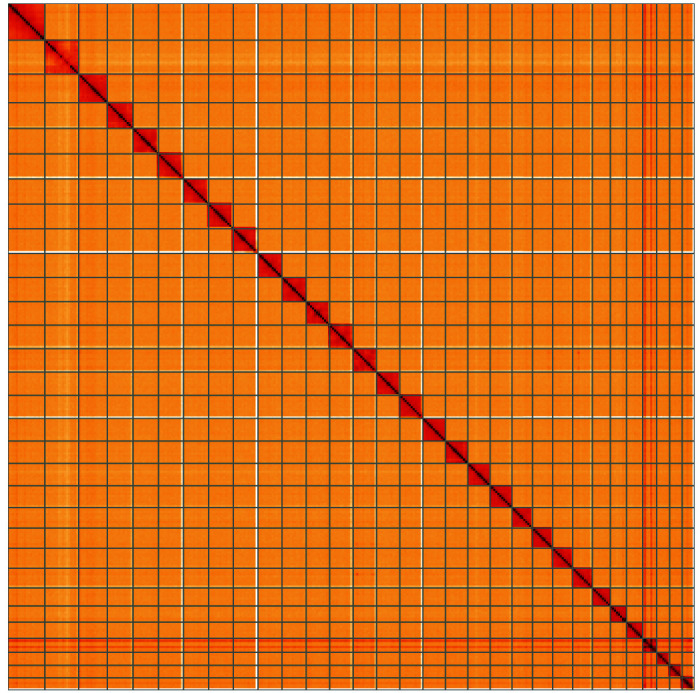
Genome assembly of *Mythimna albipuncta*, ilMytAlbi1.1: Hi-C contact map of the ilMytAlbi1.1 assembly, visualised using HiGlass. Chromosomes are shown in order of size from left to right and top to bottom. An interactive version of this figure may be viewed at https://genome-note-higlass.tol.sanger.ac.uk/l/?d=CJQyXHvuRnOqIjJo41kPdQ.

The final assembly has a total length of 698.6 Mb in 33 sequence scaffolds with a scaffold N50 of 23.9 Mb ( Table 1). The snailplot in Figure 2 provides a summary of the assembly statistics, while the distribution of assembly scaffolds on GC proportion and coverage is shown in Figure 3. The cumulative assembly plot in Figure 4 shows curves for subsets of scaffolds assigned to different phyla. Most (99.98%) of the assembly sequence was assigned to 31 chromosomal-level scaffolds, representing 30 autosomes and the Z sex chromosome. Chromosome-scale scaffolds confirmed by the Hi-C data are named in order of size ( Figure 5; Table 2). While not fully phased, the assembly deposited is of one haplotype. Contigs corresponding to the second haplotype have also been deposited. The mitochondrial genome was also assembled and can be found as a contig within the multifasta file of the genome submission.

**Table 1.** Genome data for *Mythimna albipuncta*, ilMytAlbi1.1.

Project accession data		
Assembly identifier	ilMytAlbi1.1	
Species	*Mythimna albipuncta*	
Specimen	ilMytAlbi1	
NCBI taxonomy ID	987983	
BioProject	PRJEB48329	
BioSample ID	SAMEA8603191	
Isolate information	ilMytAlbi1, male: thorax (DNA sequencing), head (Hi-C sequencing)	
Assembly metrics *		*Benchmark*
Consensus quality (QV)	62.5	*≥ 50*
*k*-mer completeness	100.0%	*≥ 95%*
BUSCO **	C:99.1%[S:98.6%,D:0.4%], F:0.2%,M:0.8%,n:5,286	*C ≥ 95%*
Percentage of assembly mapped to chromosomes	99.98%	*≥ 95%*
Sex chromosomes	Z	*localised homologous pairs*
Organelles	Mitochondrial genome: 15.38 kb	*complete single alleles*
Raw data accessions		
PacificBiosciences SEQUEL II	ERR7221641, ERR7221642	
10X Genomics Illumina	ERR7220439, ERR7220441, ERR7220440, ERR7220442	
Hi-C Illumina	ERR7220438	
Genome assembly		
Assembly accession	GCA_929112965.1	
*Accession of alternate haplotype*	GCA_929113785.1	
Span (Mb)	698.6	
Number of contigs	41	
Contig N50 length (Mb)	23.7	
Number of scaffolds	33	
Scaffold N50 length (Mb)	23.9	
Longest scaffold (Mb)	37.43	
Genome annotation		
Number of protein-coding genes	13,679	
Number of non-coding genes	3,250	
Number of gene transcripts	25,614	

* Assembly metric benchmarks are adapted from column VGP-2020 of “Table 1: Proposed standards and metrics for defining genome assembly quality” from ( Rhie *et al.*, 2021). ** BUSCO scores based on the lepidoptera_odb10 BUSCO set using version 5.3.2. C = complete [S = single copy, D = duplicated], F = fragmented, M = missing, n = number of orthologues in comparison. A full set of BUSCO scores is available at https://blobtoolkit.genomehubs.org/view/CAKMYI01/dataset/CAKMYI01/busco.

**Table 2.** Chromosomal pseudomolecules in the genome assembly of *Mythimna albipuncta*, ilMytAlbi1.

INSDC accession	Chromosome	Length (Mb)	GC%
OV815959.1	1	34.47	38.0
OV815960.1	2	29.17	38.0
OV815961.1	3	26.07	37.5
OV815962.1	4	25.94	38.0
OV815963.1	5	25.53	38.0
OV815964.1	6	25.48	37.5
OV815965.1	7	25.14	38.0
OV815966.1	8	25.13	38.0
OV815967.1	9	24.47	37.5
OV815968.1	10	24.43	38.0
OV815969.1	11	23.99	37.5
OV815970.1	12	23.91	38.0
OV815971.1	13	23.8	38.5
OV815972.1	14	23.73	37.5
OV815973.1	15	23.42	38.0
OV815974.1	16	22.97	38.0
OV815975.1	17	22.73	38.0
OV815976.1	18	22.68	38.0
OV815977.1	19	22.21	38.5
OV815978.1	20	20.96	38.0
OV815979.1	21	20.51	38.0
OV815980.1	22	20.31	38.5
OV815981.1	23	19.9	38.5
OV815982.1	24	18.43	38.5
OV815983.1	25	16.46	38.5
OV815984.1	26	16.34	38.5
OV815985.1	27	14.37	39.5
OV815986.1	28	13.19	39.5
OV815987.1	29	12.66	39.5
OV815988.1	30	12.63	40.0
OV815958.1	Z	37.43	37.5
OV815989.1	MT	0.02	19.5

The estimated Quality Value (QV) of the final assembly is 62.5 with *k*-mer completeness of 100.0%, and the assembly has a BUSCO v5.3.2 completeness of 99.1% (single = 98.6%, duplicated = 0.4%), using the lepidoptera_odb10 reference set ( *n* = 5,286).

Metadata for specimens, barcode results, spectra estimates, sequencing runs, contaminants and pre-curation assembly statistics are given at https://links.tol.sanger.ac.uk/species/987983.

## Genome annotation report

The *Mythimna albipuncta* genome assembly (GCA_929112965.1) was annotated using the Ensembl rapid annotation pipeline ( Table 1; https://rapid.ensembl.org/Mythimna_albipuncta_GCA_929112965.1/Info/Index). The resulting annotation includes 25,614 transcribed mRNAs from 13,679 protein-coding and 3,250 non-coding genes.

## Methods

### Sample acquisition and nucleic acid extraction

A male *Mythimna albipuncta* (specimen ID Ox000960, ToLID ilMytAlbi1) was collected from Wytham Woods, Oxfordshire (biological vice-county Berkshire), UK (latitude 51.77, longitude –1.34) on 2020-09-08 using a light trap. The specimen was collected and identified by Douglas Boyes (University of Oxford) and snap-frozen on dry ice.

The workflow for high molecular weight (HMW) DNA extraction at the Wellcome Sanger Institute (WSI) includes a sequence of core procedures: sample preparation; sample homogenisation, DNA extraction, fragmentation, and clean-up. In sample preparation, the ilMytAlbi1 sample was weighed and dissected on dry ice ( Jay *et al.*, 2023). Tissue from the thorax was homogenised using a PowerMasher II tissue disruptor ( Denton *et al.*, 2023a). HMW DNA was extracted using the Automated MagAttract v1 protocol ( Sheerin *et al.*, 2023). The DNA was sheared into an average fragment size of 12–20 kb in a Megaruptor 3 system with speed setting 30 ( Todorovic *et al.*, 2023). Sheared DNA was purified by solid-phase reversible immobilisation ( Strickland *et al.*, 2023): in brief, the method employs a 1.8X ratio of AMPure PB beads to sample to eliminate shorter fragments and concentrate the DNA. The concentration of the sheared and purified DNA was assessed using a Nanodrop spectrophotometer and Qubit Fluorometer and Qubit dsDNA High Sensitivity Assay kit. Fragment size distribution was evaluated by running the sample on the FemtoPulse system.

Protocols developed by the WSI Tree of Life core laboratory have been deposited on protocols.io ( Denton *et al.*, 2023b).

### Sequencing

Pacific Biosciences HiFi circular consensus and 10X Genomics read cloud DNA sequencing libraries were constructed according to the manufacturers’ instructions. DNA sequencing was performed by the Scientific Operations core at the WSI on Pacific Biosciences SEQUEL II (HiFi) and Illumina NovaSeq 6000 (10X) instruments. Hi-C data were also generated from head tissue of ilMytAlbi1 using the Arima2 kit and sequenced on the Illumina NovaSeq 6000 instrument.

### Genome assembly, curation and evaluation

Assembly was carried out with Hifiasm ( Cheng *et al.*, 2021) and haplotypic duplication was identified and removed with purge_dups ( Guan *et al.*, 2020). One round of polishing was performed by aligning 10X Genomics read data to the assembly with Long Ranger ALIGN, calling variants with FreeBayes ( Garrison & Marth, 2012). The assembly was then scaffolded with Hi-C data ( Rao *et al.*, 2014) using SALSA2 ( Ghurye *et al.*, 2019). The assembly was checked for contamination and corrected as described previously ( Howe *et al.*, 2021). Manual curation was performed using HiGlass ( Kerpedjiev *et al.*, 2018) and Pretext ( Harry, 2022). The mitochondrial genome was assembled using MitoHiFi ( Uliano-Silva *et al.*, 2023), which runs MitoFinder ( Allio *et al.*, 2020) or MITOS ( Bernt *et al.*, 2013) and uses these annotations to select the final mitochondrial contig and to ensure the general quality of the sequence.

A Hi-C map for the final assembly was produced using bwa-mem2 ( Vasimuddin *et al.*, 2019) in the Cooler file format ( Abdennur & Mirny, 2020). To assess the assembly metrics, the *k*-mer completeness and QV consensus quality values were calculated in Merqury ( Rhie *et al.*, 2020). This work was done using Nextflow ( Di Tommaso *et al.*, 2017) DSL2 pipelines “sanger-tol/readmapping” ( Surana *et al.*, 2023a) and “sanger-tol/genomenote” ( Surana *et al.*, 2023b). The genome was analysed within the BlobToolKit environment ( Challis *et al.*, 2020) and BUSCO scores ( Manni *et al.*, 2021; Simão *et al.*, 2015) were calculated.

Table 3 contains a list of relevant software tool versions and sources.

**Table 3.** Software tools: versions and sources.

Software tool	Version	Source
BlobToolKit	4.0.7	https://github.com/blobtoolkit/blobtoolkit
BUSCO	5.3.2	https://gitlab.com/ezlab/busco
FreeBayes	1.3.1-17-gaa2ace8	https://github.com/freebayes/freebayes
gEVAL	N/A	https://geval.org.uk/
Hifiasm	0.15.3	https://github.com/chhylp123/hifiasm
HiGlass	1.11.6	https://github.com/higlass/higlass
Long Ranger ALIGN	2.2.2	https://support.10xgenomics.com/genome-exome/ software/pipelines/latest/advanced/other-pipelines
Merqury	MerquryFK	https://github.com/thegenemyers/MERQURY.FK
MitoHiFi	2	https://github.com/marcelauliano/MitoHiFi
PretextView	0.2	https://github.com/wtsi-hpag/PretextView
purge_dups	1.2.3	https://github.com/dfguan/purge_dups
SALSA	2.2	https://github.com/salsa-rs/salsa
sanger-tol/genomenote	v1.0	https://github.com/sanger-tol/genomenote
sanger-tol/readmapping	1.1.0	https://github.com/sanger-tol/readmapping/tree/1.1.0

### Genome annotation

The Ensembl gene annotation system ( Aken *et al.*, 2016) was used to generate annotation for the *Mythimna albipuncta* assembly (GCA_929112965.1). Annotation was created primarily through alignment of transcriptomic data to the genome, with gap filling via protein-to-genome alignments of a select set of proteins from UniProt ( UniProt Consortium, 2019).

### Wellcome Sanger Institute – Legal and Governance

The materials that have contributed to this genome note have been supplied by a Darwin Tree of Life Partner. The submission of materials by a Darwin Tree of Life Partner is subject to the **‘Darwin Tree of Life Project Sampling Code of Practice’**, which can be found in full on the Darwin Tree of Life website here. By agreeing with and signing up to the Sampling Code of Practice, the Darwin Tree of Life Partner agrees they will meet the legal and ethical requirements and standards set out within this document in respect of all samples acquired for, and supplied to, the Darwin Tree of Life Project.

Further, the Wellcome Sanger Institute employs a process whereby due diligence is carried out proportionate to the nature of the materials themselves, and the circumstances under which they have been/are to be collected and provided for use. The purpose of this is to address and mitigate any potential legal and/or ethical implications of receipt and use of the materials as part of the research project, and to ensure that in doing so we align with best practice wherever possible. The overarching areas of consideration are:

• Ethical review of provenance and sourcing of the material

• Legality of collection, transfer and use (national and international) 

Each transfer of samples is further undertaken according to a Research Collaboration Agreement or Material Transfer Agreement entered into by the Darwin Tree of Life Partner, Genome Research Limited (operating as the Wellcome Sanger Institute), and in some circumstances other Darwin Tree of Life collaborators.

## Data Availability

European Nucleotide Archive: *Mythimna albipuncta* (white-point). Accession number PRJEB48329; https://identifiers.org/ena.embl/PRJEB48329 ( Wellcome Sanger Institute, 2022). The genome sequence is released openly for reuse. The *Mythimna albipuncta* genome sequencing initiative is part of the Darwin Tree of Life (DToL) project. All raw sequence data and the assembly have been deposited in INSDC databases. Raw data and assembly accession identifiers are reported in Table 1.

## Author information

Members of the University of Oxford and Wytham Woods Genome Acquisition Lab are listed here: https://doi.org/10.5281/zenodo.7125292.

Members of the Darwin Tree of Life Barcoding collective are listed here: https://doi.org/10.5281/zenodo.4893703.

Members of the Wellcome Sanger Institute Tree of Life Management, Samples and Laboratory team are listed here: https://doi.org/10.5281/zenodo.10066175.

Members of Wellcome Sanger Institute Scientific Operations: Sequencing Operations are listed here: https://doi.org/10.5281/zenodo.10043364.

Members of the Wellcome Sanger Institute Tree of Life Core Informatics team are listed here: https://doi.org/10.5281/zenodo.10066637.

Members of the Tree of Life Core Informatics collective are listed here: https://doi.org/10.5281/zenodo.5013541.

Members of the Darwin Tree of Life Consortium are listed here: https://doi.org/10.5281/zenodo.4783558.
