# Supplementary material for: The genome sequence of the White-point, Mythimna albipuncta (Denis & Schiffermüller, 1775)
Source: Wellcome Open Res. 2024 Feb 19;9:62. [Version 1] doi: 10.12688/wellcomeopenres.20682.1 (PMC11193084; doi:10.12688/wellcomeopenres.20682.1)
Supplement: Peer review history

# Reviewer report: Reviewer response for version 1

**DOI:** 10.21956/wellcomeopenres.22887.r85046

**Published:** 2024-07-05

**Authors:** Pasi Rastas

**Affiliations:** 1 University of Helsinki, Helsinki, Finland

The authors present a genome assembly of the White-point ( *Mythimna albipuncta*) moth. 

 Together with the normal genome assembly statistics, a brief background on the species is provided.

 The contig assembly is constructed with hifiasm software on PacBio HiFi long reads and then further polished, scaffolded and corrected by 10X Genomics and Hi-C data.

 The assembly is mostly in 31 pseudochromosomes and is of high quality. In addition, the gene annotation is provided using the Ensembl gene annotation system. 

 As minor improvement, it could mention what (if any) transcriptome data was used in the gene annotation.

Are sufficient details of methods and materials provided to allow replication by others?

Yes

Is the rationale for creating the dataset(s) clearly described?

Yes

Are the datasets clearly presented in a useable and accessible format?

Yes

Are the protocols appropriate and is the work technically sound?

Yes

Reviewer Expertise:

Genome assemblies of various species, including butterflies and moths.

I confirm that I have read this submission and believe that I have an appropriate level of expertise to confirm that it is of an acceptable scientific standard.

# Reviewer report: Reviewer response for version 1

**DOI:** 10.21956/wellcomeopenres.22887.r85049

**Published:** 2024-07-05

**Authors:** Beatriz Navarro-Domínguez

**Affiliations:** 1 Universidad de Granada, Granada, Andalusia, Spain

Boyes *et al*. have completed the genome assembly and annotation of the White-point moth ( *Mythimna albipuncta*). After providing a background on the biology and recent natural history of the White-point moth, they describe in detail their standard protocol for genome assembly, which is consistent with other reports from the Darwin's Tree of Life project. They provide details on the sampling, protocols, and resulting assemblies. The resulting assembly, which is high-quality, complete, and contiguous, is already deposited in public databases.

 I only need clarification on the below points:

- Both chromosome number and genome size vary dramatically in Lepidoptera (1,2). I have not found previous estimations of genome size, so how was the coverage needed for sequencing determined?
- I also did not find any karyotype description for this species. If only one male was sequenced, how can the authors identify the Z sex chromosome?

Are sufficient details of methods and materials provided to allow replication by others?

Yes

Is the rationale for creating the dataset(s) clearly described?

Yes

Are the datasets clearly presented in a useable and accessible format?

Yes

Are the protocols appropriate and is the work technically sound?

Yes

Reviewer Expertise:

Evolutionary genomics; Genome evolution; Selfish DNA

I confirm that I have read this submission and believe that I have an appropriate level of expertise to confirm that it is of an acceptable scientific standard.

## References

[1] : Speciation through chromosomal fusion and fission in Lepidoptera. *Philos Trans R Soc Lond B Biol Sci* .2020;375(1806) : 10.1098/rstb.2019.0539 20190539 10.1098/rstb.2019.0539 32654638 PMC7423279

[2] : Comparative genomics reveals the dynamics of chromosome evolution in Lepidoptera. *Nat Ecol Evol* .2024;8(4) : 10.1038/s41559-024-02329-4 777-790 10.1038/s41559-024-02329-4 38383850 PMC11009112

# Reviewer report: Reviewer response for version 1

**DOI:** 10.21956/wellcomeopenres.22887.r81882

**Published:** 2024-06-21

**Authors:** Annabel Whibley

**Affiliations:** 1 Grapevine Improvement, Bragato Research Institute, Lincoln, New Zealand; 2 The University of Auckland, Auckland, Auckland, New Zealand

Boyes, Holland and colleagues present the genome assembly and annotation of the White-point moth ( *Mythimna albipuncta*). The report follows the DtOL template, with systematic and comprehensive reporting of protocols, sample and assembly properties and metadata and appropriate deposition of resources in public databases. The assembly is a high-quality resource, with excellent contiguity, completeness and accuracy properties. The natural history background is knowledgeable and engaging and this genomic resource will be of value to the scientific community.

 Minor comments:

 I would prefer that the location collection co-ordinates be given their longitude and latitude qualifiers (they are in the methods, but not in the initial genome sequence report).

 I have repeatedly raised in these Data Note reviews that I believe there is an error in the PB-AMPure bead ratio reported for the clean-up of sheared DNA. In my understanding this is a 0.6x ratio of beads to sample volume, not 1.8x as stated in the template.

 I would also prefer to see the k-mer size used in MerquryFK estimates of genome properties noted, along with some details of other non-default parameter settings, for example in mapping HiC reads with BWA, though I will acknowledge that this information will be contained within the Zenodo Nextflow  workflow archives.

Are sufficient details of methods and materials provided to allow replication by others?

Yes

Is the rationale for creating the dataset(s) clearly described?

Yes

Are the datasets clearly presented in a useable and accessible format?

Yes

Are the protocols appropriate and is the work technically sound?

Yes

Reviewer Expertise:

Bioinformatics, Genomics, Evolution

I confirm that I have read this submission and believe that I have an appropriate level of expertise to confirm that it is of an acceptable scientific standard.

# Reviewer report: Reviewer response for version 1

**DOI:** 10.21956/wellcomeopenres.22887.r81888

**Published:** 2024-05-14

**Authors:** Kuppusamy Sivasankaran

**Affiliations:** 1 Loyola College, Chennai, Tamil Nadu, India

The genome of the White-point *Mythimna albipuncta* (Denis & Schiffermuller, 1775) was sequenced with appropriate techniques and assembled using standard software. Through the genome annotation authors have identified protein-coding genes, non-coding genes and gene transcripts.

 Some minor comments

 The authors have given the genus name *Mythimna albipuncta *full form throughout the article. First time the genus name should be given in full form then subsequently can be given in short form like *M. albipuncta* in the entire article.

 Table number and title for table 3 was given in between the text in page number 9. I think it may be incorrect. The table number and title can be deleted or replaced. 

 In the background of the manuscript third paragraph first line after comma “overwintering at the larval stage” is not giving proper meaning at end of the sentence. It can be rewritten.

 Last sentence of the fourth paragraph in the background can be modified as “The complete genome sequence will contribute to the growing set of resources for phylogenomic analysis in the order Lepidoptera”.

 Above all, I confirm that the manuscript meets the necessary scientific standard and is suitable for indexing"

Are sufficient details of methods and materials provided to allow replication by others?

Yes

Is the rationale for creating the dataset(s) clearly described?

Yes

Are the datasets clearly presented in a useable and accessible format?

Yes

Are the protocols appropriate and is the work technically sound?

Yes

Reviewer Expertise:

Phylogenetic analysis of Noctuoidea moth

I confirm that I have read this submission and believe that I have an appropriate level of expertise to confirm that it is of an acceptable scientific standard.
